# Degradation of Cellular miR-27 by a Novel, Highly Abundant Viral Transcript Is Important for Efficient Virus Replication *In Vivo*

**DOI:** 10.1371/journal.ppat.1002510

**Published:** 2012-02-09

**Authors:** Lisa Marcinowski, Mélanie Tanguy, Astrid Krmpotic, Bernd Rädle, Vanda J. Lisnić, Lee Tuddenham, Béatrice Chane-Woon-Ming, Zsolt Ruzsics, Florian Erhard, Corinna Benkartek, Marina Babic, Ralf Zimmer, Joanne Trgovcich, Ulrich H. Koszinowski, Stipan Jonjic, Sébastien Pfeffer, Lars Dölken

**Affiliations:** 1 Max von Pettenkofer-Institute, Ludwig-Maximilians-University Munich, Munich, Germany; 2 Architecture et Réactivité de l'ARN, Université de Strasbourg, Institut de Biologie Moléculaire et Cellulaire du CNRS, Strasbourg, France; 3 Department of Histology and Embryology, Faculty of Medicine University of Rijeka, Rijeka, Croatia; 4 Institute for Informatics, Ludwig-Maximilians-University Munich, Munich, Germany; 5 Department of Virology, Hannover Medical School, Hannover, Germany; 6 Department of Pathology, The Ohio State University, Columbus, Ohio, United States of America; 7 Department of Medicine, University of Cambridge, Addenbrooke's Hospital, Cambridge, United Kingdom; Duke University Medical Center, United States of America

## Abstract

Cytomegaloviruses express large amounts of viral miRNAs during lytic infection, yet, they only modestly alter the cellular miRNA profile. The most prominent alteration upon lytic murine cytomegalovirus (MCMV) infection is the rapid degradation of the cellular miR-27a and miR-27b. Here, we report that this regulation is mediated by the ∼1.7 kb spliced and highly abundant MCMV m169 transcript. Specificity to miR-27a/b is mediated by a single, apparently optimized, miRNA binding site located in its 3′-UTR. This site is easily and efficiently retargeted to other cellular and viral miRNAs by target site replacement. Expression of the 3′-UTR of m169 by an adenoviral vector was sufficient to mediate its function, indicating that no other viral factors are essential in this process. Degradation of miR-27a/b was found to be accompanied by 3′-tailing and -trimming. Despite its dramatic effect on miRNA stability, we found this interaction to be mutual, indicating potential regulation of m169 by miR-27a/b. Most interestingly, three mutant viruses no longer able to target miR-27a/b, either due to miRNA target site disruption or target site replacement, showed significant attenuation in multiple organs as early as 4 days post infection, indicating that degradation of miR-27a/b is important for efficient MCMV replication *in vivo*.

## Author Summary

MicroRNAs are small, non-coding RNAs which shape and fine-tune gene expression of at least a third of our genes. During millions of years of coevolution with their hosts, herpesviruses have both usurped the host cell miRNA machinery by expressing their own sets of miRNAs, and learned to modify host miRNA expression for their own needs. Recently, we reported on the rapid degradation of two cellular miRNAs upon lytic murine cytomegalovirus (MCMV) infection, namely miR-27a and miR-27b. In this paper, we show that their regulation is mediated by the highly abundant viral transcript m169. It targets miR-27a/b *via* a single binding site in its 3′-UTR, which can be efficiently retargeted to other cellular and viral miRNAs, enabling the efficient knock-down of individual miRNAs of interest. Degradation of miR-27a/b is preceded by its 3′-tailing and -trimming. Most interestingly, three mutant viruses unable to target miR-27a/b showed significantly lower virus titers in various organs during acute MCMV infection, indicating that degradation of miR-27a/b is important for efficient virus replication *in vivo*.

## Introduction

MicroRNAs (miRNAs) are small non-coding RNA molecules, which are involved in a broad range of biological processes. They represent an evolutionary highly conserved mechanism present in virtually all multicellular organisms ranging from fungi to mammals (reviewed in [1]). MiRNA biogenesis is a stepwise process involving the sequential action of the RNase III enzymes Drosha and Dicer, generating a ∼22 nucleotide (nt) miRNA duplex. One strand of the duplex is then loaded into the RNA-induced silencing complex (RISC), while the other strand, known as the passenger strand or star sequence (miRNA*), is often, but not always, actively degraded. Upon assembly into the RISC complex, which invariably contains a member of the Argonaute protein family, they regulate gene expression either *via* translational inhibition, and/or destabilization of the targeted transcript. To date, more than 1,400 miRNAs have been identified in humans [2]. Once incorporated into RISC, the loaded miRNA is thought to be rather stable, with a half-life in the range of days [3].

In the last few years, tremendous progress has been made regarding the functional role of miRNA-mediated regulation of gene expression, resulting in the identification of thousands of miRNA target sites [4]–[6]. However, much less is known about the regulation of small RNAs themselves. Regulation of miRNA expression levels has been described to occur at the level of transcription, processing, and stability (reviewed in [1]). Nevertheless, the underlying molecular mechanisms are not always clearly understood. As such, it has been reported that the regulation of the maturation step of the let-7 miRNA precursor is subject to regulation *via* the interaction of Lin28 with its terminal loop. After binding to the pre-miRNA, Lin28 recruits the terminal uridyltransferase Zcchc11, which mediates tailing of the 3′ end of the small RNA [7]–[9]. The modification of small RNAs by nucleotide addition is not only observed for pre-miRNAs, mature miRNAs can also be modified. This was initially reported in the plant model *Arabidopsis thaliana*, whereby miRNAs are usually methylated at their 3′ end by the methyl transferase HEN1 [10]. In a HEN1 mutant background, the absence of the 2′-O-methyl group on small RNAs triggers their 3′ end tailing, usually by uridylation, to bring about their degradation [11]. It has thus been proposed that the 2′-O-methylation of plant small RNAs, which also occurs on animal Piwi-interacting RNAs (piRNAs) [12], [13], actually functions to protect the small RNAs against uridylation and subsequent degradation. Recently, it was described that the interaction between a small RNA and its target can in some cases (extensive base pairing) also result in the degradation of the miRNA [14]. Interestingly, this was found to be accompanied by 3′-tailing and -trimming of the miRNA. However, the molecular mechanisms involved still remain to be identified.

Regulation of miRNA expression occurs in response to both biotic and abiotic stresses [15]. Among the former, viral infections represent a prominent part and are known to interact extensively with the RNA silencing machinery. In mammals, this is best exemplified by the hijacking of the miRNA machinery by viruses, especially herpesviruses, to express their own miRNAs [16]. The use of small, non-immunogenic RNA molecules to regulate their own, as well as cellular gene expression, is yet another illustration of the extensive adaptation of these viruses to their hosts - the result of millions of years of coevolution [17]. In addition to expressing their own miRNAs, viruses also interact with cellular miRNAs. For example, miR-32 plays an antiviral role upon primate foamy virus infection [18], and both miR-24 and miR-93 negatively regulate the vesicular stomatitis virus [19]. A recent study revealed a number of cellular miRNAs with apparently intrinsic antiviral properties [20], highlighting the need for viruses to engage in the alteration of the cellular miRNA profile. It is therefore not surprising that the expression levels of some cellular miRNAs are deregulated during viral infection.

Recently, we showed that lytic mouse cytomegalovirus (MCMV) infection, besides expressing large numbers of viral miRNAs [21], results in the dramatic destabilization of two cellular miRNAs, namely miR-27a and miR-27b [22]. MiR-27a is encoded within a miRNA cluster located on chromosome 8, along with miR-23a and miR-24-2. It possesses an isoform located on chromosome 13, miR-27b that is clustered with miR-23b and miR-24-1. Strikingly, only the level of the mature forms of miR-27a and 27b were affected upon MCMV infection, while the levels of miR-27a* and miR-27b* remained unaltered. We thus hypothesized MCMV to encode a transcript targeting miR-27a/b for rapid degradation by a yet to be identified molecular mechanism. Interestingly, during the course of this work Cazalla *et al.* identified the herpesvirus saimiri HSUR1 transcript to bind to, and target miR-27a/b for degradation [23].

Here, we report on the identification of the MCMV transcript, encoded by the m169 gene, which mediates the rapid degradation of both miR-27a and 27b. We found this down-regulation to be accompanied by 3′-tailing and -trimming of the miRNA. Specificity to miR-27a/b is mediated *via* a single binding site located in the m169 3′-UTR. Replacement of this target site allowed for efficient retargeting of the transcript to other cellular and viral miRNAs. Despite its dramatic effect on miRNA stability, we found this interaction to be mutual, resulting in miR-27a/b-mediated regulation of m169. We thus performed infections of mice with the mutant viruses we generated, which lost the ability to degrade miR-27a/b, but retained regulation by a retargeted cellular or viral miRNA. Results from these experiments reveal that the interplay between the m169 transcript and cellular miRNAs is important during acute MCMV infection *in vivo*.

## Results

### Degradation of miR-27a/b is mediated by a single binding site in the MCMV m169 transcript

**Figure 1:**
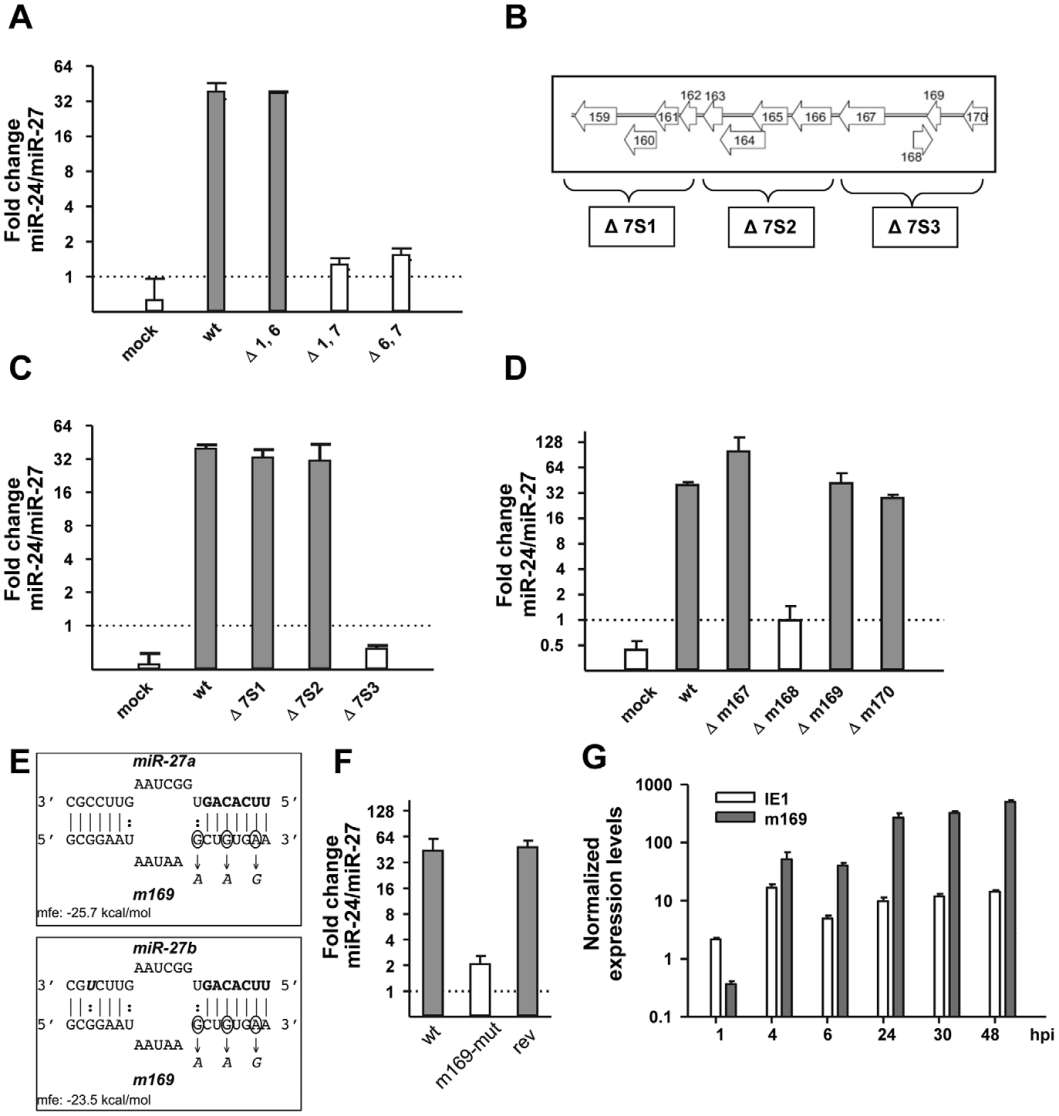
MCMV m169 transcript is targeting miR-27 *via* a single binding site in its predicted 3′-UTR. **A**. Gene block 7 (m149–m170) is crucial for miR-27 degradation. NIH-3T3 cells were infected with wild-type MCMV or three large deletion mutants each lacking two of the three gene blocks 1 (m01–m16), 6 (m144–m158) or 7 (m159–m170) at an MOI of 10. 48 h post infection total RNA was isolated and miR-27 levels were quantified relative to miR-24 by qRT-PCR. **B**. Genomic organization of gene block 7 and the three subdividing mutants. **C**. Degradation of miR-27 is mediated by a gene located in between m167 and m170. The effect of the indicated mutants on miR-27 expression was determined as described above. **D**. The genomic region encompassing the m168 coding sequence is essential for degradation of miR-27. The effect of the indicated mutants on miR-27 expression was determined as described above. This implicated either m168 or m169 to be implicated in miR-27 degradation. **E**. The binding site of m169/miR-27a and miR-27b interactions. RNAhybrid predicted a single, well defined binding site for miR-27a/b in the predicted 3′-UTR of the m169 transcript showing a typical match-bulge-match structure. The three point mutations introduced by markerless mutagenesis to create MCMV-m169-mut are indicated. Only the middle one truly affects this interaction due to G:U 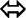 A:U base pairing altered by the other two. In addition, the binding site to miR-27b is shown with the single nucleotide difference to miR-27a indicated in bold italics. The mean free energy (mfe) of the interactions was determined using RNAhybrid. **F**. Mutagenesis of the binding site abolished miR-27 degradation. The effect of the mutant virus and its revertant on miR-27 levels were determined as described above. **G**. Expression levels of m169 and IE1 transcripts. NIH-3T3 fibroblasts were infected with WT MCMV at an MOI of 10. At the indicated time points of infection total RNA was prepared. Expression levels of m169 and IE1 were determined by qRT-PCR and normalized for Lamin B receptor (LBR) mRNA levels.

Upon lytic infection with the murine cytomegalovirus (MCMV), large amounts of viral miRNAs are rapidly produced [21], [24]. Recently, we reported that in addition to the expression of its own viral miRNAs, MCMV infection triggers the rapid degradation of two mature cellular miRNAs in various cell types, namely miR-27a and miR-27b, [22]. These two cellular miRNAs show complete sequence identity but for the nineteenth nucleotide (“C” for miR-27a and “U” for miR-27b). As both miRNAs appeared to be similarly affected by MCMV, and because both quantitative RT-PCR (qRT-PCR) and northern blot analysis cannot properly distinguish miR-27a from miR-27b, we will refer indiscriminately to both miRNAs as miR-27. In detail, their degradation presented as a measureable decrease of the two mature miRNAs within 4 hours of infection, ultimately leading to an almost complete disappearance of miR-27 within two days of infection. Blocking viral gene expression in MCMV infected cells prevented miR-27 degradation [22]. It was thus tempting to speculate that this massive, apparently sequence-specific degradation of two cellular miRNAs was mediated by a viral transcript binding to the mature miRNAs, and subsequently targeting them for degradation *via* a yet to be discovered molecular mechanism. We decided to test this hypothesis by screening large deletion mutants to identify the gene responsible for this function. We started off with three MCMV mutants (Δ1,6; Δ1,7; Δ6,7) that we previously generated [25], each lacking two of the three gene blocks encompassing either MCMV genes m1–m16 (block 1), m144–m159 (block 6) or m159–m170 (block 7). It is important to note that none of these mutants shows any attenuation on NIH-3T3 fibroblasts *in vitro*, while all three show severe growth defects *in vivo* [25]. In order to ensure efficient infection of >99% of all cells, we always infected NIH-3T3 fibroblast with a multiplicity of infection (MOI) of 10 for 48 h, and determined changes in miR-27 expression levels relative to miR-24 by quantitative RT-PCR (qRT-PCR). We chose miR-24 for normalization as it is expressed from the same pri-miRNA transcript as miR-27. In addition, we found qRT-PCR C**_T_** values for miR-24 to be virtually identical to miR-27 in uninfected cells, thus providing an ideal mean for normalization using the ΔΔC**_T_** -method. In accordance with our previous observations [22], wild-type MCMV infection resulted in a ∼30-fold reduction in miR-27 levels compared to miR-24 (Figure 1A). While miR-27 degradation was still apparent with the Δ1,6 mutant, both mutants lacking gene block 7 (m159–m170) completely lost their ability to target miR-27 for degradation. Therefore, degradation of miR-27 indeed appeared to be mediated by a viral gene product. We thus generated and tested a second set of smaller deletion mutants (Δ7S1, Δ7S2 and Δ7S3, see Figure 1B), each lacking four MCMV genes of gene block 7. The results obtained with these three mutant viruses, following the same experimental setup as before, pinpointed the responsible viral gene to m167–m170 (Figure 1C). Finally, we generated and tested a third set of mutants (Δm167, Δm168, Δm169 and Δm170) lacking the predicted coding sequences (CDS) of the corresponding genes. Only the mutant lacking the predicted m168 CDS was no longer able to target miR-27 for degradation (Figure 1D). We thus looked for a putative binding site for miR-27 on either strand of the genome within the 389 nt deletion of m168 using RNA-hybrid [26]. Interestingly, this revealed a single binding site on the strand opposite to m168, within the predicted 3′-untranslated region (UTR) of m169. The binding site consists of a perfect match to the first 7 nt of miR-27a (6-mer seed match), a G-U pairing at the 8^th^ position, a 5 *vs.* 6 nt bulge (known to prevent target degradation *via* Ago2 slicer activity), and a 7 nt perfect match to the 3′-end of the miRNA (including one G-U pairing adjacent to the bulge) (Figure 1E). The one nucleotide difference between miR-27a and miR-27b only results in a change from an A-U to a G-U pairing, which was predicted by RNA-hybrid to render the interaction to m169 slightly weaker for miR-27b than for miR-27a (mean free energy (mfe) = −23.5 kcal/mol *vs.* −25.7 kcal/mol). Markerless mutagenesis to introduce three single point mutations into the seed region of the predicted binding site (MCMV-m169-mut) (marked in Figure 1E) resulted in an almost complete loss of miR-27 degradation from ∼30-fold, down to ∼2-fold (Figure 1F). Degradation of miR-27 was completely restored for the revertant virus. However, it is important to note that only the middle one of these three point mutations actually disrupts seed pairing, as both A-U and G-U pairings are known to support RNA-RNA interactions, thus rendering the other two mutations less potent. Therefore, a G-U wobble at position 2, and a single mismatch in the middle of the seed region were sufficient to almost completely abolish miR-27 degradation.

To get further insights into the characteristics of the m169 transcript, we made use of data generated by the Jonjic and Trgovcich laboratories, which performed a large scale cDNA cloning and sequencing study to comprehensively characterize the MCMV transcriptome (Lisnic *et al.*, manuscript in preparation). In this study, a ∼1.7 kb transcript containing the m169 open reading frame (ORF) was identified (for transcript details see Figure S1). This analysis also revealed a novel intron of 78 nt, located close to the 3′-end of the putative m169 CDS. The miR-27 binding site is located 104 nt 3′ of the m169 stop codon at the 5′ end of the m169 transcript 3′-UTR. Quantitative RT-PCR revealed this transcript to be already expressed at high levels within the first hour of infection, exceeding IE1 transcripts by >2-fold within 4 hours of infection, and >20-fold at 48 hours post infection (hpi) (Figure 1G).

### Degradation of miR-27 by the m169 transcript involves its 3′-tailing and –trimming

**Figure 2:**
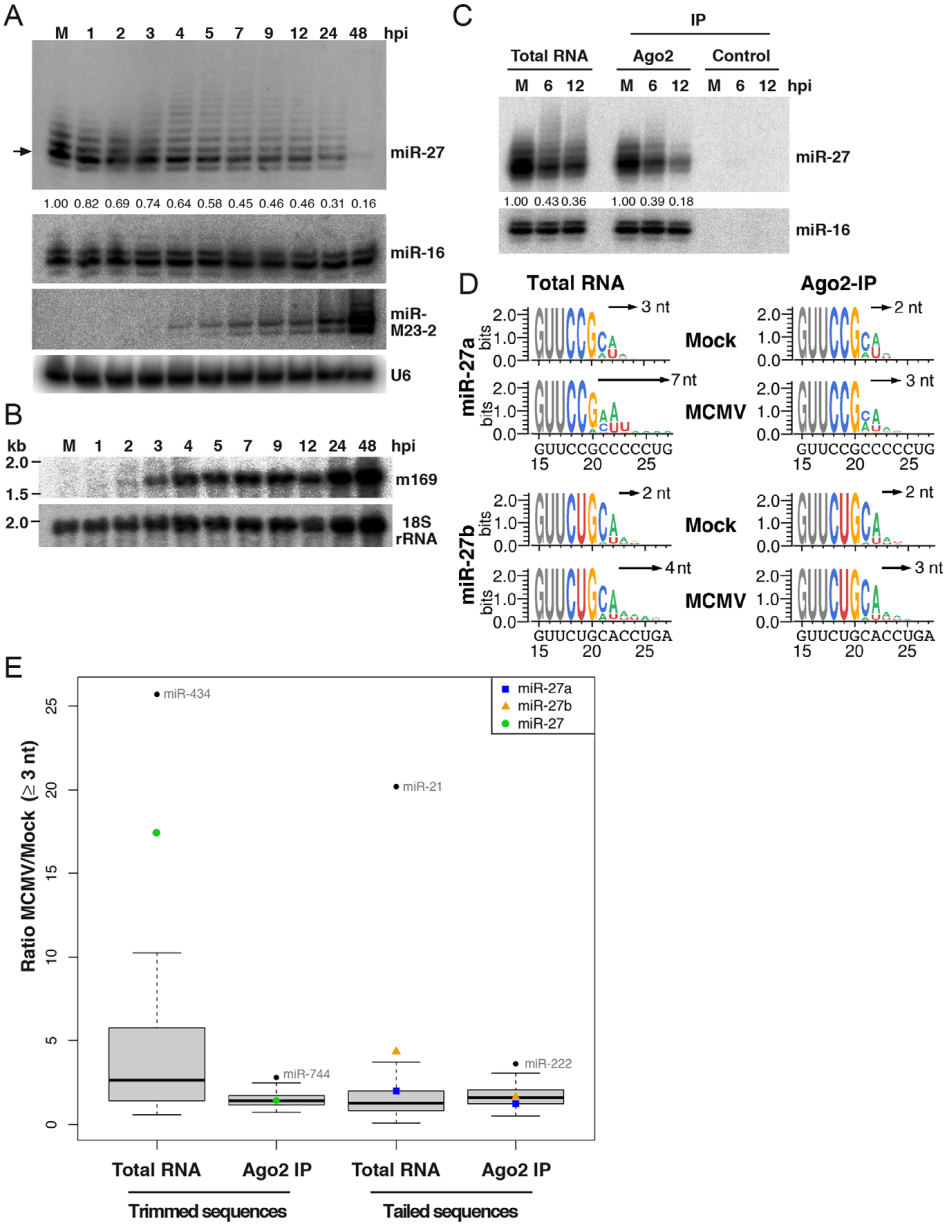
Degradation of miR-27 is linked to its 3′ -tailing and -trimming. **A**. Northern blot analysis of miR-27, miR-16, miR-M23-2 in NIH-3T3 cells infected with wild type MCMV (MOI = 10). The arrow indicates the prominent band representing the mature miR-27, which is quantified relative to Mock (M) as indicated by the numbers below the blot. U6 snRNA was used as a loading control. **B**. Northern blot analysis of m169 transcript accumulation over time in the same MCMV-infected NIH-3T3 cells. 18S rRNA was used as a loading control. **C**. Accumulation of miR-27a and miR-27b in Ago2 IP and total RNA in mock (M) and from cells infected with MCMV for 6 and 12 hours. Signals were normalized to miR-16 and quantified relative to mock-infected cells. **D**. Sequence logo representations for tailed miR-27a and 27b retrieved from deep-sequencing analysis of total RNA or RNA extracted after Ago2 IP of mock or MCMV-infected cells (6 hpi). Only nucleotides 15 to 27 of miR-27 sequences presenting 3′ addition are represented, nucleotides of interest are depicted in colour, and the sizes of the largest tails are indicated next to the arrows. The genomic sequences of pre-miR-27a and b are indicated underneath the sequence logo representations. **E**. Boxplot representation of the trimmed and tailed MCMV/mock sequence ratios for the most abundant miRNAs shared by each library. Only sequences presenting with a 3′-trimming or a -tailing of ≥3 nt in total RNA or RNA extracted after Ago2 IP were considered. The outliers are indicated by black dots and miR-27a and b are indicated by a blue square and an orange triangle respectively. The green dot indicates sequences that could not be annotated as either miR-27a or 27b due to their trimming. The black bar indicates the median of all ratios.

Recently, it was reported that extensive complementarity between a target RNA and a miRNA can result in post-transcriptional modifications of the miRNA, mainly addition of nucleotides at the 3′ end of the small RNA (tailing), and shortening of the sequence from its 3′ extremity (trimming) [14]. We therefore looked for post-transcriptional modifications of miR-27 at early times of infection prior to its degradation. We infected NIH-3T3 cells with wild-type MCMV at an MOI of 10 and collected cells to extract total RNA every hour from 1 to 5 hpi, and at 7, 9, 12, 24 and 48 hpi. We then performed high-resolution small RNA northern blot analysis on these RNA samples and probed for miR-27, miR-16 as well as the viral mcmv-miR-M23-2. As can be seen on Figure 2A, miR-27 accumulated as four distinct bands in the mock infected cells. During the first two to three hours of MCMV infection, the pattern of miR-27 bands did not change, but after four hours of infection additional bands started to appear, while conjointly the expression level of the major band started to decrease. The number of extra-bands visible on the miR-27 blot culminated at 7 hpi before gradually decreasing again, the quantification of the most prominent band showed that miR-27 was almost completely degraded by 48 hpi. The level of the control miRNA miR-16 was not affected throughout the time course, and the accumulation of the mcmv-miR-M23-2 occurred as expected [21]. At the same time, we also measured the accumulation of the viral transcript m169 and showed that it started to become detectable by northern blot at 2 hpi, and was abundantly expressed at 4 hpi, coinciding with the appearance of additional bands in the miR-27 blot (Figure 2B).

In order to gain further insight into the level at which the degradation of miR-27 occurs, we then performed Ago2 immunoprecipitation (IP) of NIH-3T3 cells infected with MCMV at 6 and 12 hpi. We analyzed the accumulation of miR-27 in both the total RNA and RNA isolated after Ago2 IP. Although the resolution of this gel was not as good as in the previous northern blot, we could still see some tailing of miR-27 in the total RNA at 6 and 12 hpi (Figure 2C). Interestingly, the extent of tailing seemed to be lower in the RNA isolated from the Ago2 IP. In addition, miR-27 levels decreased significantly more rapidly in the Ago2 IP than in total RNA (Figure 2C), indicating that tailing of miR-27 might be accompanied by its displacement from Ago2 complexes subsequently followed by its degradation, or that extensive tailing occurred after displacement of miR-27 from Ago2.

We then set up to determine the nature of the modification of miR-27a and miR-27b by performing small RNA cloning and Illumina deep-sequencing using either total RNA extracted from mock-infected NIH-3T3 cells, or from cells infected with MCMV for 6 hours, as well as from RNA extracted after Ago2 IP from the same samples. The analysis of miR-27a and miR-27b sequences in the libraries revealed that in both mock- and MCMV-infected cells, addition of non-templated nucleotides could be detected at the 3′ end, consistent with the northern blot data. Therefore, the additional bands seen for miR-27 by northern blot represent 3′-tailing with non-templated nucleotides, rather than processing isoforms. However, it is important to note that this feature was not unique to miR-27, but also seen for other cellular miRNAs. As can be seen in a sequence logo representation [27], [28] of cloned miR-27a and b in total RNA extracted from MCMV-infected cells, the number of added nucleotides was greater for miR-27a (up to 3 nt in the mock *vs.* 7nt in the infected cells) than miR-27b (2 nt in mock *vs.* 4 nt MCMV infected cells respectively) (Figure 2D). The number of sequences presenting with longer tails, however, was lower for miR-27a than for miR-27b (Tables S1 and S2). The most prominent nucleotide added was an ‘A’, followed by a ‘U’. Intriguingly, in Ago2 IP the difference in tailing between mock and MCMV infected cells was neither evident for miR-27a nor miR-27b, indicative of displacement of extensively tailed miRNAs from Ago2, or conversely, tailing of miRNAs after displacement from Ago2. To estimate the significance of the modifications of miR-27 in MCMV infected cells, we extracted (for the most abundantly cloned cellular miRNAs) the number of sequences presenting with a tail of ≥3 nt, and calculated the ratio of these values between MCMV and mock infected cells. Given that miRNA tailing has been associated with their trimming, we also performed the same analysis for sequences trimmed by at least 3 nt. We found that, in total RNA, miR-27b was significantly more tailed in MCMV infected cells compared to mock infected cells (4.35-fold) than any other cellular miRNA (median of ratio = 1.59) (Figure 2E). The ratio for miR-27a was 2.00, which is above the mean, but still inside the interquartile range. In addition, the enrichment of tailed miR-27b in MCMV infected cells was lost in Ago2 IP (Figure 2E). Only one other cellular miRNA, namely miR-21, showed significantly enhanced 3′ tailing in MCMV infected cells (Figure 2E). As for miR-27b, this was only seen in total RNA but not in the Ago2 IP. Regarding the trimmed miRNAs, a strong enrichment (17.64-fold *vs.* a median ratio of 2.63) in MCMV infected cells compared to mock infected cells could be seen for miR-27 (due to the trimming, these sequences could not be attributed to either miR-27a or b). As for the tailed sequences, this enrichment was lost in the Ago2 IP. Only one other outlier, namely miR-434, showed an increased trimming in total RNA of MCMV *vs.* mock infected cells. Interestingly, this miRNA was also observed to be down-regulated in MCMV-infected cells (data not shown). We were unable to resolve trimming of miR-27 by northern blot. Finally, we also looked at miRNAs tailed or trimmed with ≥1 nt (Figure S2A), or ≥2 nt (Figure S2B), but did not find a significant difference between MCMV infected cells and non-infected cells for miR-27a and b. This is most probably due to the fact that both miRNAs already show the addition of up to 2 nucleotides in mock-infected cells, thereby introducing substantial variability and thus noise to our analysis. A global overview of all modifications observed for miR-27a and b can be found in Table S3.

### Retargeting of m169 to other cellular and viral miRNAs by target site replacement

**Figure 3:**
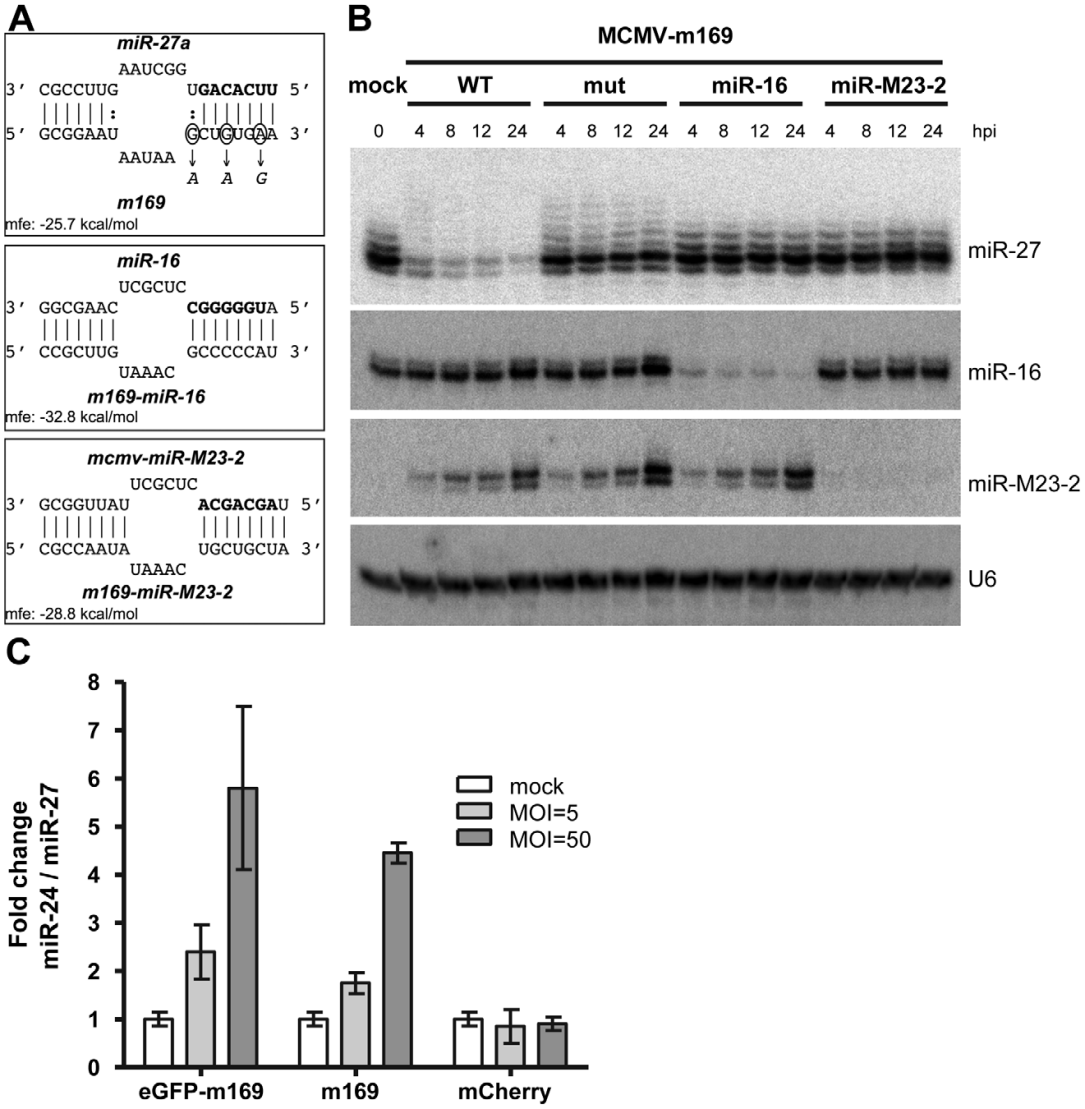
Retargeting of m169 to cellular and viral miRNAs. **A**. Schematic representation of the miR-27a/m169 binding site and the introduced mutated binding sites resulting in retargeting of m169 to either mmu-miR-16 or mcmv-miR-M23-2. Target site predictions were performed using RNAhybrid to design the retargeting of m169. **B**. Efficient retargeting of m169 to a cellular and a viral miRNA. NIH-3T3 cells were infected with the indicated viruses and total RNA isolated at different times of infection was analyzed by northern blot for miR-27, miR-16 and mcmv-miR-M23-2. U6 served as loading control. Retargeting of m169 to miR-16 and mcmv-miR-M23-2 abolished degradation of miR-27 but resulted in highly efficient degradation of the respective miRNAs. **C**. Expression of m169 3′-UTR is sufficient to mediate miR-27 degradation. Adenoviral vectors expressing either full-length m169 or its 3′-UTR fused to EGFP were used to transduce NIH-3T3 cells at an MOI of either 50 or 500. An adenoviral vector expressing mCherry was used as negative control. Two days post-transduction, total RNA was prepared and miR-24/miR-27 ratios were determined by qRT-PCR.

In order to further characterize the mechanism of miR-27 degradation and obtain useful tools for further studies, we next tested whether we could retarget m169 to other cellular or viral miRNAs. We thus replaced the m169 binding site for miR-27 with a binding site for the ubiquitous cellular miR-16, or for the highly abundant viral miRNA mcmv-miR-M23-2 (Figure 3A). Binding sites were designed to mimic the natural match-bulge-match structure of the miR-27a binding site. We chose mcmv-miR-M23-2 as we recently found this miRNA (together with mcmv-miR-21-1) to be required for viral persistence in salivary glands [29]. After generating the mutant viruses (MCMV-m169-miR-16 and MCMV-m169-miR-M23-2) we performed a time-course of infection in NIH-3T3 cells with these viruses and performed northern blot analysis to probe for the effect of their retargeting on miR-27, miR-16 and mcmv-miR-M23-2 levels (Figure 3B). As expected, retargeting of m169 to other miRNAs by altering the miR-27 binding site completely abolished the ability of both mutants to mediate miR-27 degradation. In contrast, the MCMV-m169-mut still showed a slight reduction of miR-27 levels consistent with the ∼2-fold reduction in miR-27 levels observed by qRT-PCR (see Figure 1F). Apparently, the single G-C to A-C change and the two A-U 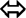 G-U changes to the seed region of the m169 binding site were not sufficient to completely abolish miR-27 binding. Most interestingly, this was accompanied by enhanced tailing of miR-27, represented by the appearance of additional bands on the northern blot. In contrast, complete disruption of this binding site by retargeting it to either miR-16 or mcmv-miR-M23-2 did not show this phenomenon. Together with the sequencing data shown in Figure 2D, these data provide strong evidence that miR-27 binding at this site mediates its tailing, either directly, or by displacing it from its Argonaute protein.

Infection with MCMV-m169-miR-16 resulted in the rapid degradation of miR-16 within the first four hours of infection. In addition, infection with MCMV-m169-miR-M23-2 completely abolished any detectable signal for mcmv-miR-M23-2 despite its strong expression and continuous accumulation during lytic MCMV infection (Figure 3B). Therefore, target site replacement of this single miRNA binding site in m169 is sufficient to efficiently target both cellular and viral miRNAs for rapid degradation.

### Expression of m169 transcript alone is sufficient to target miR-27 for degradation

To test whether any other viral factors were involved in miR-27 degradation, we generated replication-deficient adenoviral vectors expressing either full-length m169 or its 3′-UTR fused to EGFP controlled by an EF1 promoter. An adenoviral vector expressing mCherry served as negative control. NIH-3T3 cells were transduced using an MOI of 50 or 500 (virus titers determined using HEK-293 cells) in the presence of TransMAX reagent to enhance transduction efficiency. Forty-eight hours after transduction of NIH-3T3 cells with these four adenoviruses, miR-27, miR-24 and m169 transcript levels were determined by qRT-PCR. Interestingly, expression of the 3′-UTR of m169 fused to EGFP was sufficient to mediate an MOI-dependent ∼6-fold loss of miR-27 levels (Figure 3C). This is in accordance with m169 transcript levels being the highest in this condition but still 5- to 10-fold lower than those achieved by lytic MCMV infection as determined by qRT-PCR (data not shown). Therefore, expression of the 3′-UTR of m169 fused to a protein coding gene is sufficient to target a cellular miRNA for degradation and no other viral factors appear to be essential to this process.

As the 3′-UTR of m169 was apparently sufficient to mediate degradation of miR-27, we looked for additional motifs in it. We noticed a uridine-rich stretch located 69 nt 3′ of the miR-27 binding site. Using markerless mutagenesis we mutated this stretch from ‘UUUUUGUUUUUU’ to ‘AAGAAGAAAGAA’ in the MCMV genome and reconstituted the mutant virus. However, this did not alleviate degradation of miR-27 following high MOI infection of NIH-3T3 fibroblasts (Figure S3).

### m169 is a target of miR-27

**Figure 4:**
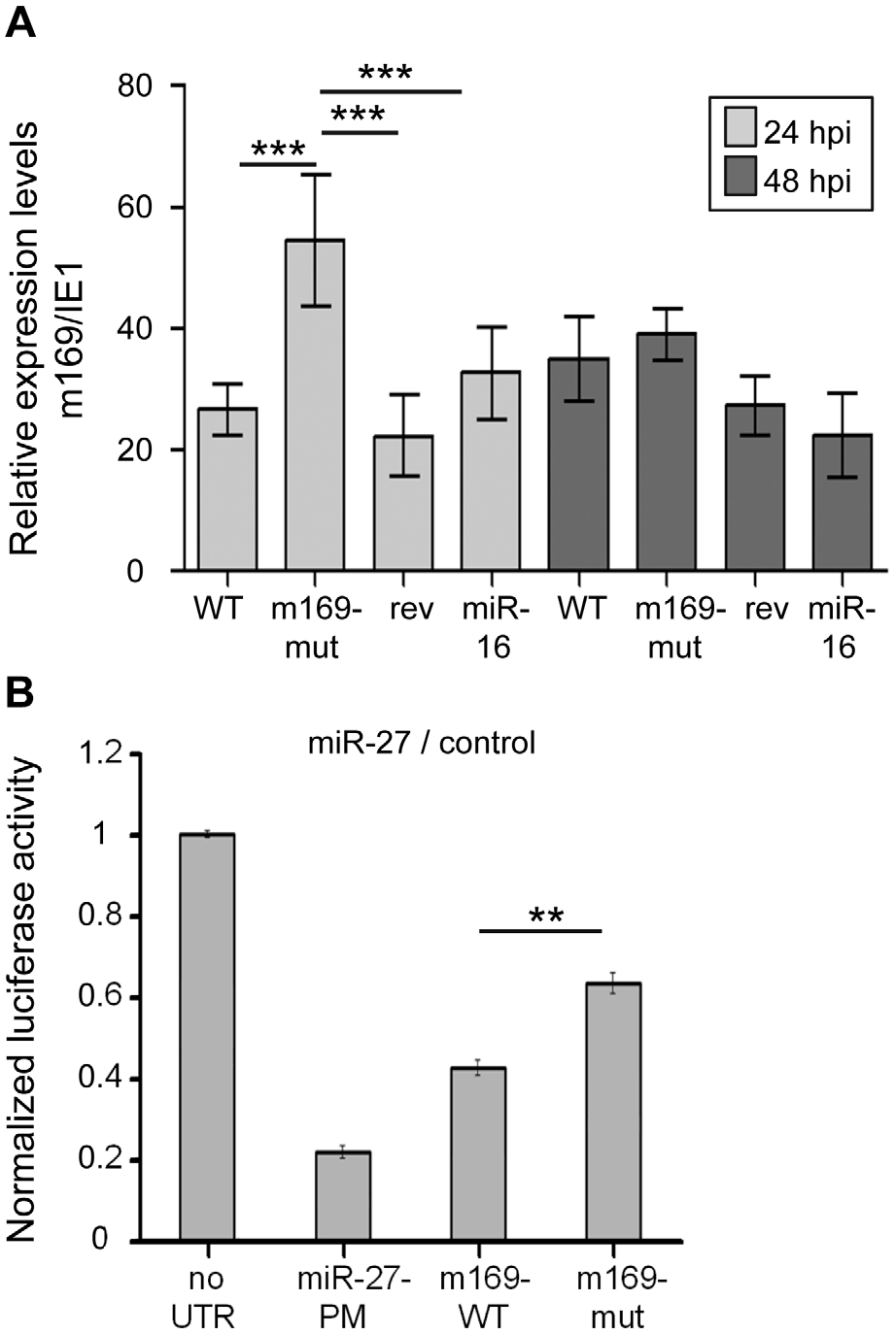
m169 is a target of miR-27. **A**. Effect on m169 RNA levels. To assess the effect of the miRNA/m169 interaction on m169 transcript levels, NIH-3T3 cells were infected with wild-type MCMV, MCMV-m169-mut, its revertant as well as the retargeted mutant MCMV-m169-miR-16. At 24 and 48 hpi total RNA was prepared and m169 transcript levels were determined by qRT-PCR. In parallel IE1 RNA levels were quantified to normalize for dose of infection. Disruption of the miR-27 binding site (MCMV-m169-mut) resulted in significantly increased (One-way anova analysis followed by Tukey's multiple comparisons test: ***: p<0.0001) m169 transcript levels at 24 hpi. This was restored when m169 was retargeted to miR-16 and no longer seen at 48 hpi. **B**. Dual-luciferase assay with psiCHECK2 constructs containing either no UTR, a perfect match (PM) binding site for miR-27, the wild type or the point mutant version of the m169 3′UTR. HeLa WS cells were co-transfected with the luciferase construct and a control miRNA mimic, or a miR-27a mimic. The ratios of normalized luciferase values for miR-27 and control mimics are indicated. ** p<0.001.

While the m169 transcript obviously has a detrimental impact on miR-27 or any other miRNA binding to it in a miR-27-like manner, we needed to consider the option that the virus might simply utilize an abundant cellular miRNA to regulate expression of one of its genes. We thus investigated the effect of miR-27 on m169 RNA levels. First, we looked at m169 RNA levels following infection with wild-type MCMV, MCMV-m169-mut, its revertant, as well as the retargeted mutant MCMV-m169-miR-16. Expression levels of m169 mRNA at 24 and 48 hpi were determined relative to IE1 mRNA levels (to normalize for differences in infection) using qRT-PCR. The level of m169 mRNA was significantly greater (∼2-fold, p<0.001) for MCMV-m169-mut than for the other three viruses (Figure 4A). This is consistent with a partial loss of regulation of m169-mut by miR-27 (resulting in increased m169 levels) and an efficient retargeting of m169 by miR-16 which we found to be expressed at very similar levels as miR-27 using qRT-PCR (data not shown). At 48 hpi no differences in m169 transcript levels were observed anymore.

We next assessed whether a luciferase sensor containing the m169 3′-UTR could be regulated by miR-27. We therefore generated pSI-CHECK2 plasmids containing either a perfect match to miR-27, the full-length m169 3′UTR, or the point mutant version of the 3′ UTR that was used in the virus infection experiment. These constructs were co-transfected into HeLa cells with control or miR-27 miRNA mimics (to prevent any possible down-regulation of the miRNA by these targets). As can be seen in Figure 4B, the perfect match sensor for miR-27 was 80% down-regulated by miR-27, while the m169 3′-UTR reporter showed a 60% down-regulation. The insertion of the three point mutations in the m169 3′-UTR only partially alleviated its regulation by miR-27 to about 40%, which is consistent with the 2-fold effect of the respective mutant virus on miR-27 levels at 48 hpi. This indicates that the m169-mut transcript can no longer efficiently mediate miR-27 degradation, but is still partially regulated by miR-27. In summary, these data indicate a complex mutual interaction between miR-27 and m169.

### The reciprocal interaction between miR-27 and m169 is important for *in vivo* infection

**Figure 5:**
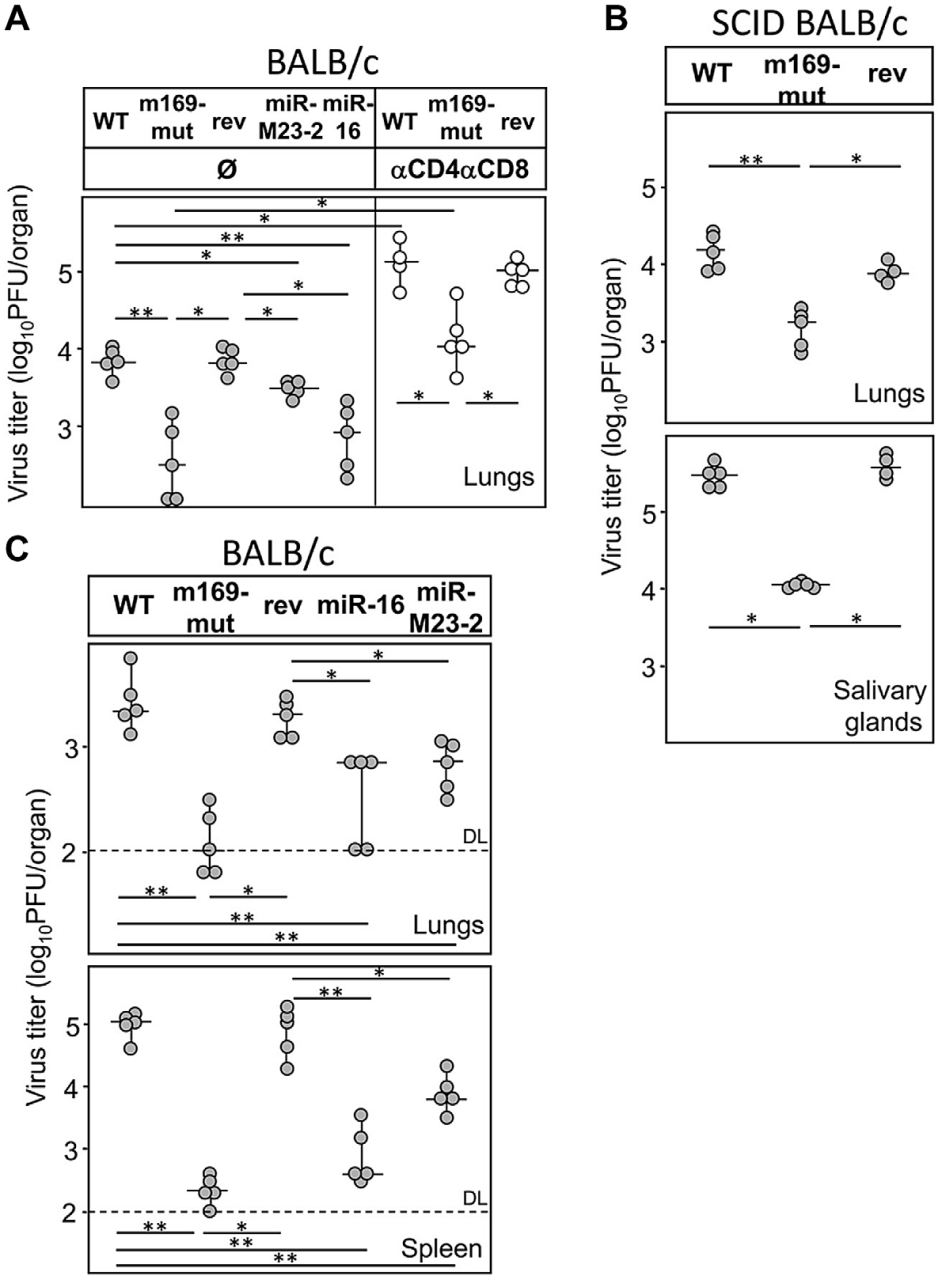
Degradation of miR-27 is important for efficient virus replication *in vivo*. BALB/c (**A**) or SCID BALB/c (**B**) mice were injected intravenously (*i.v.*) with 1×10^5^ PFU of wild-type (wt), MCMV-m169-mut, m169-mut-rev or the two retargeted viruses MCMV-m169-miR-16 and –miR-M23-2. Some of the BALB/c mice were depleted of CD4 and CD8 T cells. At 14 dpi organs were harvested and virus titers in lungs and salivary glands determined by standard plaque assay. **C**. BALB/c mice were injected *i.v.* with 1×10^5^ PFU of indicated viruses. At 4 dpi organs were harvested and virus titers in lungs and spleen determined by standard plaque assay. Viral titers in organs of individual mice (circles) and median values (horizontal bars) are shown. DL = detection limit; * p<0.05; ** p<0.01.

As the interaction between the m169 transcript and miR-27a appears to be mutual, the key question was whether the virus uses the m169 transcript to target an important cellular miRNA for degradation, or whether it simply uses an irrelevant but highly expressed cellular miRNA to reduce and delay protein expression of one of its genes. To address this question, we made use of our mutant and retargeted viruses and studied their phenotypes following 14 days of infection in mice. While we designed the three point mutations in MCMV-m169-mut such as not to alter the predicted m168 coding sequence, it is important to note that this was not possible for both MCMV-m169-miR-16 and MCMV-m169-miR-M23-2. For MCMV-m169-miR-M23-2 this altered six amino acids in m168. For MCMV-m169-miR-16 this resulted in a frame-shift and a premature stop codon 28 amino acids later (for details see Table S4). So far nothing is known about the function of m168, or whether it is expressed at all. However, we failed to detect any expression of the m168 transcript even at late time-points (both 24 and 48 hpi) of lytic MCMV infection of NIH-3T3 fibroblasts using next-generation sequencing (unpublished data). We thus infected BALB/c mice for 14 days with these viruses and determined virus titers in lungs. Interestingly, we observed a significant 3- to 12-fold attenuation of all three viruses, the greatest attenuation being observed for the m169-mut virus; no attenuation was observed for the revertant virus MCMV-m169-mut-rev (Figure 5A). This was also seen in BALB/c mice depleted for both CD4^+^ and CD8^+^ T-cells, indicating that the observed attenuation was independent of T-cell function. Next, we infected SCID BALB/c mice with wild-type MCMV, MCMV-m169-mut, or its revertant. At 14 days post infection (dpi) we again observed significant (∼10-fold) attenuation of this mutant virus in both lungs and salivary glands (Figure 5B). We conclude that the quite striking role of miR-27 during lytic MCMV infection is independent of adaptive immunity. To test whether this phenotype would become apparent even at earlier stages of acute infection, we infected BALB/c mice for four days and compared virus titers in lung and spleen. While virus titers in lungs showed only a moderate, but consistent reduction in virus titers for all three mutants no longer targeting miR-27, a strong 10- to 100-fold attenuation of all three mutants was observed in spleen (Figure 5C). Altogether, these data highlight that degradation of miR-27 by the MCMV m169 transcript, and presumably also the miRNA-mediated regulation of m169 protein expression are important factors during lytic virus infection *in vivo*.

## Discussion

Besides expressing large amounts of viral miRNAs shortly after infection [21], MCMV also targets two cellular miRNAs for rapid degradation, namely miR-27a and b [22]. Using a set of deletion mutants and site directed mutagenesis, we show this effect to be mediated by a single binding site located within the 3′-UTR of a so far uncharacterized, highly abundant, spliced MCMV transcript, namely m169. We found this effect to be readily redirected to other cellular and viral miRNAs by target site replacement. Interestingly, expression of the m169 3′-UTR fused to EGFP by an adenoviral vector was sufficient to mediate degradation of miR-27, indicating that no other viral factors are essentially required in this process.

Recently, Ameres *et al.* reported on tailing and trimming of cellular miRNAs upon extensive base-pairing to their targets [14]. However, the underlying mechanism still remains to be determined. Thus, it is not known whether this is happening to miRNAs still bound by an Argonaute protein, or after their displacement from RISC. Upon wild-type MCMV infection, we observed extensive tailing of miR-27 by northern blot within the first few hours of infection, prior to its subsequent degradation. The tailing and degradation of miR-27 was not observed in cells infected with mutant viruses in which the miR-27/m169 RNA interaction was disrupted by retargeting m169 to either miR-16 or mcmv-miR-M23-2. This argues against a general effect of MCMV infection on miR-27 tailing. Despite the point mutations we introduced into the miR-27 binding site to generate MCMV-m169-mut, the m169-mut transcript was still able to bind miR-27, demonstrated by significant miR-27-mediated repression in dual-luciferase assays. In addition, it still resulted in a somewhat reduced but significant activity for miR-27 degradation (∼2-fold instead of ∼30-fold) as observed by both qRT-PCR and northern blot. Interestingly, we observed enhanced tailing of miR-27 following infection with MCMV-m169-mut compared to wild-type MCMV infection. Most likely, this is due to the larger amounts of miR-27 still present at 6–24 hpi. While these data thus provide strong hints that binding of miR-27 to this single binding site not only triggers its degradation but also its tailing, further studies are required to clarify the link between the two.

Consistent with the northern blot data, small RNA deep-sequencing revealed 3′-tailing with 1 to 3 non-template nucleotides for both miR-27a and miR-27b in uninfected cells. The presence of non-template tailing is a common feature of miRNAs, indicating that this is not unique to miR-27a/b. This was seen in both total RNA as well as Ago2 IP samples independent of MCMV infection. Upon MCMV infection, however, we observed both an enhanced tailing of miR-27b, as well as trimming of miR-27 (≥3nt) in small RNA libraries prepared from total RNA. Interestingly, this increased tailing and trimming of miR-27 was not detectable in small RNA isolated from Ago2 IP samples, which might indicate that these modifications occur after m169-mediated displacement of miR-27 from Ago2, or that the tailed and trimmed miR-27 forms are rapidly excluded from it. This is partially corroborated by the northern blot analysis, which showed a stronger MCMV-mediated down-regulation of miR-27 in Ago2 than in total RNA. The observation that miR-27b, and not miR-27a, showed enhanced tailing might be due to its slightly weaker interaction with m169, because a Watson-Crick base pair with the m169 transcript is replaced by a G-U pairing. It is thus tempting to speculate that we simply might have cloned and sequenced more tailed miR-27b in MCMV infected cells because it is less efficiently degraded than miR-27a – thereby either leaving less time for tailing of miR-27a following displacement from Ago2, or speeding up displacement of tailed miR-27a from Ago2 followed by its more rapid degradation. This would also explain the more rapid loss of both miR-16 and mcmv-miR-M23-2 following infection with the retargeted mutants, rendering tailing of these miRNAs below the detection limit of our northern blots. Studies are ongoing to address this important issue.

In a previous study [21], we already reported on extensive tailing of two viral miRNAs, namely mcmv-miR-m21-1 and mcmv-miR-M23-2, which was accompanied by a decrease of these miRNAs late in infection. As we found these two miRNAs to derive from a genomic locus transcribed from both the sense and antisense strand, tailing and degradation of these two miRNAs might result from binding to viral transcripts expressed from the antisense strand. Together with the data presented in this study, we therefore postulate that highly expressed transcripts, such as MCMV m169, can cause miRNA tailing (and trimming) followed by their degradation.

As m169 targets Argonaute-bound miR-27 for degradation, it was very reasonable to assume that m169 may also serve as a target for miR-27. Indeed we observed significantly higher m169 RNA levels following infection with MCMV-m169-mut than for wild-type MCMV, its revertant, or MCMV-m169-miR-16. Interestingly, this was only observed at 24 hpi, which was verified in three independent experiments. In contrast, at 48 hpi when wild-type MCMV infection has efficiently eliminated miR-27, its effect on m169 expression was relieved. As such, the mutual interaction between the m169 transcript and miR-27 is apparently complex. The key question really was whether the main function of m169 is to target an important cellular miRNA for degradation, or whether the virus simply uses an irrelevant but highly expressed cellular miRNA to reduce and delay protein expression of one of its genes. With the help of our retargeted viruses we were able to partially address this important question. Interestingly, all three mutant viruses no longer able to efficiently target miR-27 for degradation were significantly attenuated at 4 and 14 dpi in all experiments we performed. This was particularly prominent in both spleen at 4 dpi, and in lungs at 14 dpi. It is, however, important to note that the generation of both MCMV-m169-miR-16 and MCMV-m169-miR-M23-2 (but not MCMV-m169-mut) resulted in alterations to the predicted m168 coding sequence. While we were unable to detect m168 expression by next-generation sequencing even at late times of infection (unpublished data; data not shown) this does not rule out that alterations in m168 may have contributed to the observed phenotype. In addition, we cannot exclude that degradation of miR-16 by MCMV-m169-miR-16 may have contributed to this phenotype. In contrast, we recently provided an extensive set of data showing that knock-out of mcmv-miR-M23-2 has no apparent effect on lytic virus replication in various organs at 4 dpi and in lungs at 14 dpi [29]. The knock-out (which also includes the knock-out of mcmv-miR-m21-1 which is generated from the antisense strand) only resulted in significantly lower virus titers in salivary gland at 14 dpi. In the course of the current study we decided to generate MCMV-m169-miR-M23-2 hoping to be able to further dissect the phenotype of the miR-m21-1/-M23-2 double miRNA knock-out virus in salivary gland [29]. However, as all three viruses no longer able to efficiently target miR-27 for degradation also showed significant attenuation in salivary gland at 14 dpi, this aim could not be achieved. In conclusion, it may thus well be that other factors than degradation of miR-27 contributed to the attenuation of the different mutants we observed. In particular the role of miR-27 in controlling the timing of m169 expression requires further studies. This and other factors are likely to have contributed to the attenuation of our three mutant viruses. However, as all three mutants deficient for miR-27 degradation were attenuated *in vivo* we believe this provides good evidence that degradation of miR-27 is indeed important during acute MCMV infection *in vivo*.

So far little is known about the function of miR-27. Our data provide strong evidence that miR-27 expression is harmful to productive MCMV infection. Interestingly, the Steitz laboratory recently showed that the oncogenic herpesvirus saimiri also uses one of its non-coding RNAs to target miR-27 for degradation [23]. This supports the interpretation of our data that degradation of miR-27 is indeed of importance for MCMV, and probably also for herpesvirus saimiri. The mechanism by which degradation of miR-27 aids lytic MCMV infection still remains elusive. To date, these two miRNAs have been implicated in pathways such as cell differentiation [30], adipogenesis [31], and angiogenesis [32]. Among the validated cellular targets, the tumor suppressor FOXO1, which is involved in controlling the cell cycle through CDKN1C induction [33], might provide a link to the strong phenotype we observed during acute MCMV infection. Indeed, cytomegaloviruses are known for their manipulation of the cell cycle [34], and m169 may act by releasing the effects of miR-27 on cell cycle progression. Interestingly, recent reports showed that the level of the miR-16 family of miRNAs are efficiently destabilized in response to cell-cycle changes [35]. Therefore, rapid alterations in cellular miRNAs are likely to be of importance in the regulation of cell cycle progression.

Why does m169 so efficiently target miR-27 for degradation? We noted m169 to be expressed at extremely high levels within 4 h of infection, exceeding levels of IE1 by >20-fold at 24 h post infection. The number of m169 transcripts obviously matters. Mutagenesis of a uridine-rich motif, which we observed in close proximity to the miR-27 binding site, had no effect on miR-27 degradation. The shift from a classical miRNA-target interaction, where the miRNA regulates its target, and the opposite effect, *i.e.* miRNA degradation mediated by its RNA target, might thus simply be controlled by the relative abundance of the target RNA. When the m169 transcript reaches a certain level, it may thus start to trigger degradation of miR-27, ultimately avoiding its regulatory effect. Further studies are required to address this interesting question.

In conclusion, there is now increasing evidence that the expression of miRNAs is strongly regulated, and that these molecules are not as stable as they were once thought to be. They are part of a sophistically tuned, complex reciprocal interaction network with the mRNA targets they regulate. The ability of cytomegalovirus to usurp the underlying cellular machinery by expressing decoy targets which bind to specific miRNAs and degrade them is yet another illustration of the fascinating dynamism of RNA-mediated regulation.

## Materials and Methods

### Ethics statement

All of the protocols used for breeding of mice and different kinds of treatments were approved by the Ethical Committee of the Faculty of Medicine University of Rijeka and were performed in accordance with Croatian Law for the Protection of Laboratory Animals, which has been harmonized with the existing EU legislation (EC Directive 86/609/EEC).

### Cell lines

BALB/c murine embryonic fibroblasts (MEFs) and M2-10B4 bone marrow stroma cells (ATCC: CRL-1972) were cultured in DMEM containing 10% fetal calf serum and penicillin/streptomycin. NIH-3T3 fibroblasts (ATCC: CRL-1658) were cultured in DMEM medium containing 5% fetal calf serum and penicillin/streptomycin.

### Generation of mutant viruses

Generation of deletion mutants to screen for the gene responsible for miR-27a/b degradation was performed as described previously [36] using the full-length MCMV BAC pSM3fr [37]. All PCR primers are included in Table S5A. Markerless BAC mutagenesis was performed to introduce more subtle changes into the MCMV m169 locus. This was performed according to previously published protocols [38], [39]. Briefly, an I-SceI-aphAI cassette was amplified from the plasmid pEPKAN-S by 2-step PCR using the different sets of primers included in Table S5B. In the first Red recombination step these PCR fragments were inserted into pSM3fr, resulting in a BAC carrying a kanamycin resistance cassette flanked by I-SceI restriction sites. The kanamycin resistance cassette was then removed from kanamycin-resistant clones by an arabinose-induced I-SceI digestion and subsequent heat-induced Red recombination, resulting in markerless mutagenesis of the miR-27a/b binding site in m169. The revertant virus (MCMV-m169-mut-rev) was generated from the m169-mut BAC by completely restoring the wild-type sequence, *i.e.* reverting all three point mutations to wild-type sequence. All mutant BACs were subjected to extensive restriction pattern analysis using at least three different restriction enzymes followed by sequencing of the altered m169 locus. All viruses were reconstituted by transfecting the recombinant BACs into murine embryonic fibroblasts and virus titers were determined on MEFs by standard plaque assay as described [36].

### Generation of adenoviruses expressing full-length or parts of m169

Adenoviral vectors expressing either full-length m169, or its 3′-UTR fused to EGFP under control of the cellular EF1-promoter were generated by Sirion Biotech, Martinsried, Germany. An adenovirus expressing mCherry served as control. To increase transduction efficiency of NIH-3T3 cells, TransMAX enhancer reagent (Sirion Biotech) was used following the manufacturer's instruction using an MOI of 50 and 500 (titers derived from infection of HEK-293 cells, corresponding to an MOI of about 5 and 50 in NIH-3T3 cells).

### Infection of NIH-3T3 cells

Six-well plates were seeded with 0.5×10^6^ cells one day prior to infection in DMEM containing 10% fetal calf serum. Cells were infected with indicated viruses at an MOI of 1 followed by centrifugal enhancement (800 g, 30 min), resulting in an effective MOI of ∼10; culture media was replaced following one hour of infection.

### Quantifying changes in miRNA levels by qRT-PCR

NIH-3T3 cells were infected with wild-type MCMV or mutant viruses at an MOI of 10. 48 h post infection RNA was prepared using either Trizol reagent (Invitrogen) or miRNeasy columns (Qiagen) following the manufacturers' instructions. cDNA was prepared in a single step reaction for both miRNAs and larger transcripts using the miScript Reverse Transcription kit (Qiagen). Light Cycler qRT-PCR was performed for various cellular and viral miRNAs using the miScript SYBR Green PCR kit (Qiagen) following the manufacturer's instructions. In each case the nucleotide sequence of the mature target miRNA was used to design the miRNA-specific forward primer. The PCR program was composed of a denaturation step at 94°C for 12 min followed by 45 cycles of 95°C for 15 sec (ramp rate 20°C/sec), 55°C for 30 sec (ramp rate 20°C/sec; 60°C for mRNA targets) and 70°C for 1 sec (ramp rate 2°C/sec). A list of all PCR primers is provided in Table S5C.

### Immunoprecipitation of mouse Argonaute 2 complexes

To determine whether the m169 transcript was recruited to Ago2-complexes, immunoprecipitation of miRNA/target complexes was performed using the recently published antibody to mouse Ago2 [40]. Per replicate, ten large (15 cm) dishes of NIH-3T3 cells were infected with an MOI of 10 for 0, 6 or 12 h with wild-type MCMV. Cells were washed twice with PBS before lysis in 10 ml lysis buffer, containing 25 mM Tris HCl pH 7.5, 150 mM KCl, 2 mM EDTA, 0.5% NP-40, 0.5 mM DTT, and Complete protease inhibitor (Roche). DTT and protease inhibitors were always prepared freshly and added immediately before use. Lysates were incubated for 30 min at 4°C and cleared by centrifugation at 20,000 g for 30 min at 4°C. Total RNA was prepared from 100 µL of cell lysates using the miRNeasy kit (Qiagen) following the manufacturer's instructions. Ago2-immunoprecipitation was performed as recently described [41]. In short, 6 µg of purified monoclonal mAgo2-antibody (2D4, Wako) or monoclonal BrdU-antibody (abcam; used as control) was added to 5 mL of RPMI-medium and incubated with 30 µL of Protein-G-Sepharose beads (GE Healthcare) in Pierce centrifuge columns (Thermo Scientific) under constant rotation at 4°C over night. Columns were drained by gravity flow and washed once with the lysis buffer. Beads were subsequently incubated with 5 mL of cell lysates for 2.5 h under constant rotation at 4°C. After incubation, the beads were washed four times with IP wash buffer (300 mM NaCl, 50 mM Tris HCl pH 7.5, 5 mM MgCl_2_, 0.1% NP-40, 1 mM NaF) and once with PBS to remove residual detergents. RNA was recovered from the beads by adding 700 µl of Qiazol to the columns. After 5 min the Qiazol lysates were collected from the columns. This step was repeated once and the Qiazol lysates were combined. RNA was prepared using the miRNeasy kit (Qiagen) according to the manufacturer's instructions. RNA samples were eluted in 30 µL of H_2_O. Efficiency of the immunoprecipitation was checked by qRT-PCR for miR-16 and let7a.

### Northern blot analysis

#### Small RNAs

RNA was extracted using Trizol and northern blotting was performed on 10 µg of total RNA. Briefly, total RNA was resolved on a 17.5% acrylamide gel of 30 cm in length. RNA was transferred by semi-dry transfer (BioRad TransBlot SD) to a Hybond-NX membrane (GE Healthcare). RNAs were then cross-linked to the membrane by EDC cross-linking as described in Pall and Hamilton [42] with a 90 min incubation step at 65°C. Prehybridization, hybridization, and wash steps were performed at 50°C. Probes either consisted of 5′-^32^P-radiolabelled oligodeoxynucleotides only or oligodeoxynucleotides containing Locked Nucleic Acid positions for the detection of tailed forms of miR-27 (Eurogentec). They are perfectly complementary to the miRNA sequence or to part of the U6 snRNA sequence.

#### m169 transcript

Total RNA samples (1 µg) were mixed with equal volumes of loading dye and heated at 65°C for 10 minutes prior to separation on a 1.2% agarose/formaldehyde gel. RNA was transferred overnight to a Hybond-NX membrane (GE Healthcare) by standard capillary transfer methods and crosslinked with UV. Membranes were prehybridized for 2 hrs in PerfectHyb™ plus (Sigma) at 50°C. Antisense DNA oligos for m169 (5′-GGACGGGGGGAGACGGCGGACGAG) and mouse 18S (5-CGGAACTACGACGGTATCTG) were 5′ end labelled using T4 polynucleotide kinase (Fermentas) with 25 µCi of [γ-32P]dATP. The labelled probe was hybridized to the blot overnight at 50°C. The blot was then washed at 50°C twice for 20 min (5× SSC/0.1% SDS), followed by an additional wash (1× SSC/0.1% SDS) for 20 min.

Northern blots were exposed to phosphorimager plates (Fuji) and scanned using a FLA-5000 series phosphorimager (Fuji).

### Small RNA cloning and sequencing

To assess the role of MCMV in the regulation of miR-27, four small RNA libraries were generated, either from total RNA, or from small RNAs immunoprecipitated with Ago2. For libraries generated from total RNA, 10 µg of total RNA was used in the initial step. For libraries generated from small RNAs incorporated into Ago2 containing RISC complexes, a fraction of the mAgo2 IPs from either MCMV infected (6 hpi), or control cells were used. Samples were size fractionated on a 17.5% PAGE gel, and small RNAs between 19 and 33 nt were excised and cloned as previously described [43], except that small RNA PCR products were not concatamerized, and instead sent directly for sequencing. Small RNA libraries were sequenced at the Institut de Génétique et de Biologie Moléculaire et Cellulaire (IGBMC, Illkirch, France) using an Illumina Genome Analyzer IIx with a read length of 36 nt.

### Deep-sequencing data analysis

Short sequences generated by the Illumina platform were pre-processed and annotated using an in-house pipeline, specifically designed to detect miRNA variants. We first applied the Dustmasker program [44] to filter out low complexity reads, before removing the 3′ adaptor from the remaining sequences using the FASTX-Toolkit (http://hannonlab.cshl.edu/fastx_toolkit). Trimmed reads of 15 to 32 nt in length were then selected to be mapped simultaneously to the mouse (UCSC repository - assembly version mm9) and the MCMV (RefSeq database - accession number NC_004065.1) genomes using Bowtie [45] by permitting up to 1 mismatch in the first 15 read nucleotides and no limit beyond. Reads that could map to more than 20 loci were discarded and a post-filtering was applied to keep only the best alignment(s) of each small RNA sequence provided this (these) alignment(s) did not exceed a total number of 9 mismatches compared to the reference genome. From there, all known mature and star miRNAs derived from *Mus musculus* and MCMV (miRBase v.17) were annotated by comparing their genomic coordinates to that of the reads, and by keeping reads with at least 50% of their length inside the genomic feature. By doing so, we were able to inventory and quantify all miRNA forms: full length, 3′-trimmed, 3′-tailed, 5′-trimmed, 5′-tailed and every possible combination (5′-trimmed+3′-trimmed, 5′-trimmed+3′-tailed, 5′-tailed+3′-trimmed and 5′-tailed+3′-tailed), may they be identical or different to the template sequence. During the quantification process, multiple mapped reads were weighted by a factor 1/n, n being the number of alignments they were respectively involved in. To determine the nature of the modifications affecting miR-27a and miR-27b upon infection, these two miRNAs were more precisely analyzed and manually curated to redistribute the few sequences that were mis-annotated between the two forms. These mis-annotations were most probably due to the poor constraints applied on the 3′ read ends during the mapping step.

The data discussed in this publication have been deposited in NCBI's Gene Expression Omnibus and are accessible through GEO Series accession number GSE34475 (http://www.ncbi.nlm.nih.gov/geo/query/acc.cgi?acc=GSE34475).

For the generation of sequence logo representations and for the comparison of libraries, sequencing data were normalized per million miRNA reads. In addition, only sequences that were cloned at least once per 1×10^6^ miRNA reads were considered to produce Figure 2D, Table S1 and Table S2. For Figure 2E and Figure S2, we first determined the 100 most abundant miRNAs in each library before selecting those that were common to all four libraries. We then extracted their respective tailed (only non-template additions were considered) and trimmed sequences, and in both cases we only kept modified miRNAs that were represented by at least 1 read per million miRNA reads in all four libraries. Finally, we calculated the sequence ratios for each selected miRNA between MCMV and mock infected libraries, and generated boxplot graphs. For Table S3, raw sequences of miR-27a and b prior to any filter were taken into account.

### Mice

BALB/c (H-2^d^) and SCID BALB/c (H-2^d^) mice were housed and bred under specific-pathogen-free conditions at the Central Animal Facility, Faculty of Medicine, University of Rijeka.

### Luciferase assays

HeLa WS cells were seeded in 48-well plates at 25,000 cells/well and then incubated for 24 h. Cells were then co-transfected using Lipofectamine 2000 (Invitrogen) with 25 ng of the reporter constructs, 250 ng of carrier pUC19 DNA and 10 nM of miRNA mimic (cel-miR-67 as a control or miR-27a). After 24 h, cells were then washed in PBS and lysed with 65 µL of passive lysis buffer (Promega), and 10 µL were assayed for Firefly and Renilla luciferase activity, using the dual-luciferase reporter assay system (Promega) and a luminescence module (Glomax, Promega). The relative reporter activity was obtained by first normalizing to the transfection efficiency with the Renilla activity, and then, to the firefly activity obtained for the empty control reporter, in presence of the miR-27 mimic or control mimic, to normalize for the effect of transfection of these oligonucleotides. Finally, the ratios of the values obtained for miR-27 and control mimics were calculated.

### Infection conditions and detection of infectious MCMV in tissues, depletion of lymphocyte subsets and statistical evaluation

Mice were injected intravenously with 1×10^5^ PFU of tissue culture-grown wild-type MCMV or recombinant viruses in 0.5 mL of diluent. Organs were collected either 4 or 14 days after infection and virus titers were determined by a standard plaque-forming assay [46]. *In vivo* depletion of B and T cells was performed by intraperitoneal injection of the mAbs to CD4 (YTS191.1), to CD8 (YTS 169.4) molecules [47]. Statistical significance of the difference between experimental groups was determined by the Mann-Whitney exact rank test.

### Accession numbers of genes

The following murine genes were mentioned in the text: Glyceraldehyde-3-phosphate dehydrogenase (GAPDH; NM_008085.1), Lamin B receptor (LBR; NM_133815.2), Interferon regulatory factor 1 (IRF1; NM_008390.2), mmu-miR-27a (MIMAT0000537), mmu-miR-27b (MIMAT0000126), mmu-miR-24 (MIMAT0000219), mmu-miR-16 (MIMAT0000527).

## Supporting Information

Figure S1 **Annotated nucleotide sequence of the m169 transcript (Smith strain).**
(TIF)

Figure S2 **Boxplot representations of the trimmed and tailed MCMV/mock sequence ratios for the most abundant miRNAs common to all libraries.** Only sequences trimmed or tailed by ≥1 nt (**A**) or ≥2 nt (**B**) in total RNA or RNA extracted after Ago2 IP were considered. The outliers are indicated by black dots and miR-27a and b are indicated by a blue square and an orange triangle, respectively. The black bar indicates the median of all ratios.
(TIF)

Figure S3 **Mutagenesis of the pyrimidine-rich stretch close to the miR-27 binding site has no significant effect on miR-27 degradation.** To generate MCMV-m169-URS-mut, the pyrimidine-rich stretch located 69 nt 3′ of the miR-27 binding site was mutated from ‘TTTTTGTTTTTT’ to ‘AAGAAGAAAGAA’ using markerless mutagenesis. The primers used to amplify the Kanamycin resistance gene encoded on the plasmid pEPKAN-S are included in Table S5. Following virus reconstitution, NIH-3T3 cells were infected with mock, m169-URS-mut or wild-type MCMV (WT) at an MOI of 10. At 48 hpi miR-24 and miR-27 levels were determined by q-RT-PCR. No significant difference in miR-27 degradation between m169-URS-mut and wild-type MCMV infection was observed.
(TIF)

Table S1 **miR-27a Sequences from total RNA and Ago2-IP used to generate** **Figure 2D** **.** Sequences that were used to generate the sequence logo representation of miR-27a in Figure 2D. The full-length sequence is indicated in grey at the top of the list, and sequences presenting with 3′ additions are listed afterward. For each sequence, the size and the normalized number of reads (per million miRNA reads) are indicated. Only sequences cloned at least once per 1×10^6^ miRNA reads were taken into account.
(XLS)

Table S2 **miR-27b Sequences from total RNA and Ago2-IP used to generate** **Figure 2D** **.** Sequences that were used to generate the sequence logo representation of miR-27b in Figure 2D. The full-length sequence is indicated in grey at the top of the list, and sequences presenting with 3′ additions are listed afterward. For each sequence, the size and the normalized number of reads (per million miRNA reads) are indicated. Only sequences cloned at least once per 1×10^6^ miRNA reads were taken into account.
(XLS)

Table S3 **Modifications (Tailing/Trimming) of miR-27a and b sequences in total RNA and Ago2-IP.** Representation of all observed modifications of miR-27a and b in the deep-sequencing libraries. fl: full length; id: identical (similar to genomic sequence); iso: isoform (differs from genomic sequence); 3trim: 3′ trimmed; 3add: 3′ tailed; 5trim: 5′ trimmed; 5add: 5′ tailed. The normalized number of reads (per million miRNA reads) is represented as well as the ratio in percentage relative to the other modifications.
(XLS)

Table S4 **Changes in nucleotide sequence of the different mutants and their impact on the putative m168 coding sequence.** Changes in nucleotide sequence and in the encoded aminoacids are shown.
(XLSX)

Table S5 **List of all primers used for PCR or cloning.** Primers used for generation of MCMV deletion mutants using ET-cloning (**A**), for the generation of MCMV mutants using Markerless mutagenesis (**B**) and for qRT-PCR (**C**) are shown.
(XLSX)
