# Supplementary figures and images for: Degradation of Cellular miR-27 by a Novel, Highly Abundant Viral Transcript Is Important for Efficient Virus Replication In Vivo
Source: PLoS Pathog. 2012 Feb 9;8(2):e1002510. doi: 10.1371/journal.ppat.1002510 (PMC3276556; doi:10.1371/journal.ppat.1002510)

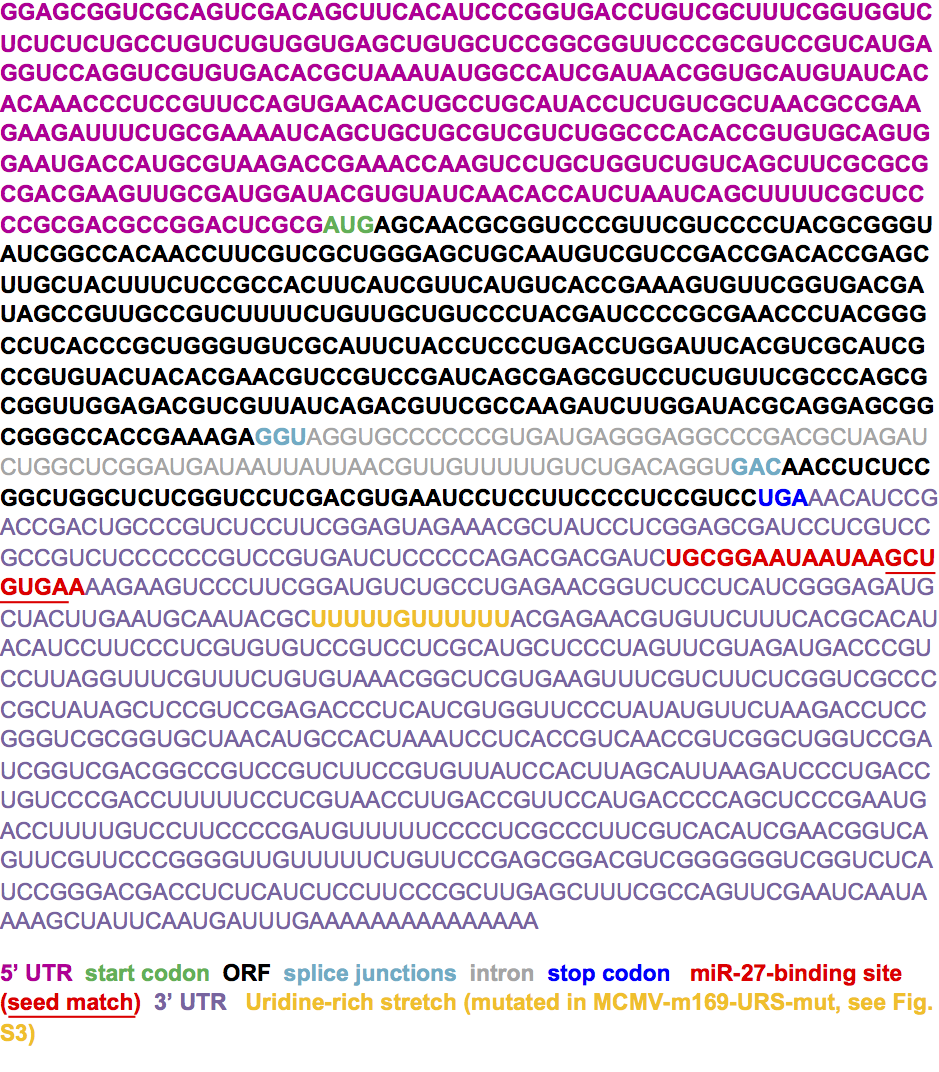

Supplement: Figure S1 — Annotated nucleotide sequence of the m169 transcript (Smith strain). (TIF) [file ppat.1002510.s001.tif]

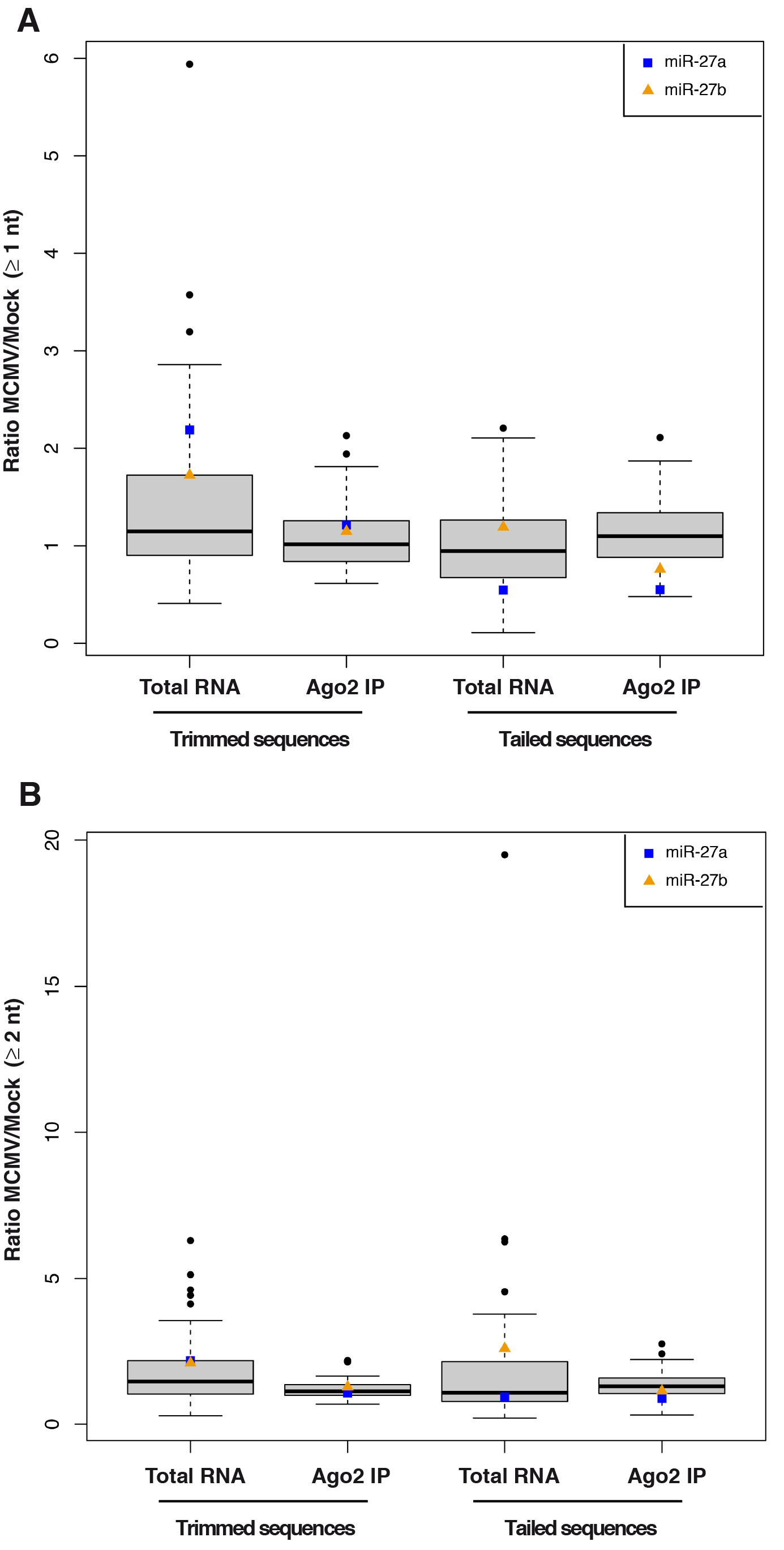

Supplement: Figure S2 — Boxplot representations of the trimmed and tailed MCMV/mock sequence ratios for the most abundant miRNAs common to all libraries. Only sequences trimmed or tailed by ≥1 nt (A) or ≥2 nt (B) in total RNA or RNA extracted after Ago2 IP were considered. The outliers are indicated by black dots and miR-27a and b are indicated by a blue square and an orange triangle, respectively. The black bar indicates the median of all ratios. (TIF) [file ppat.1002510.s002.tif]

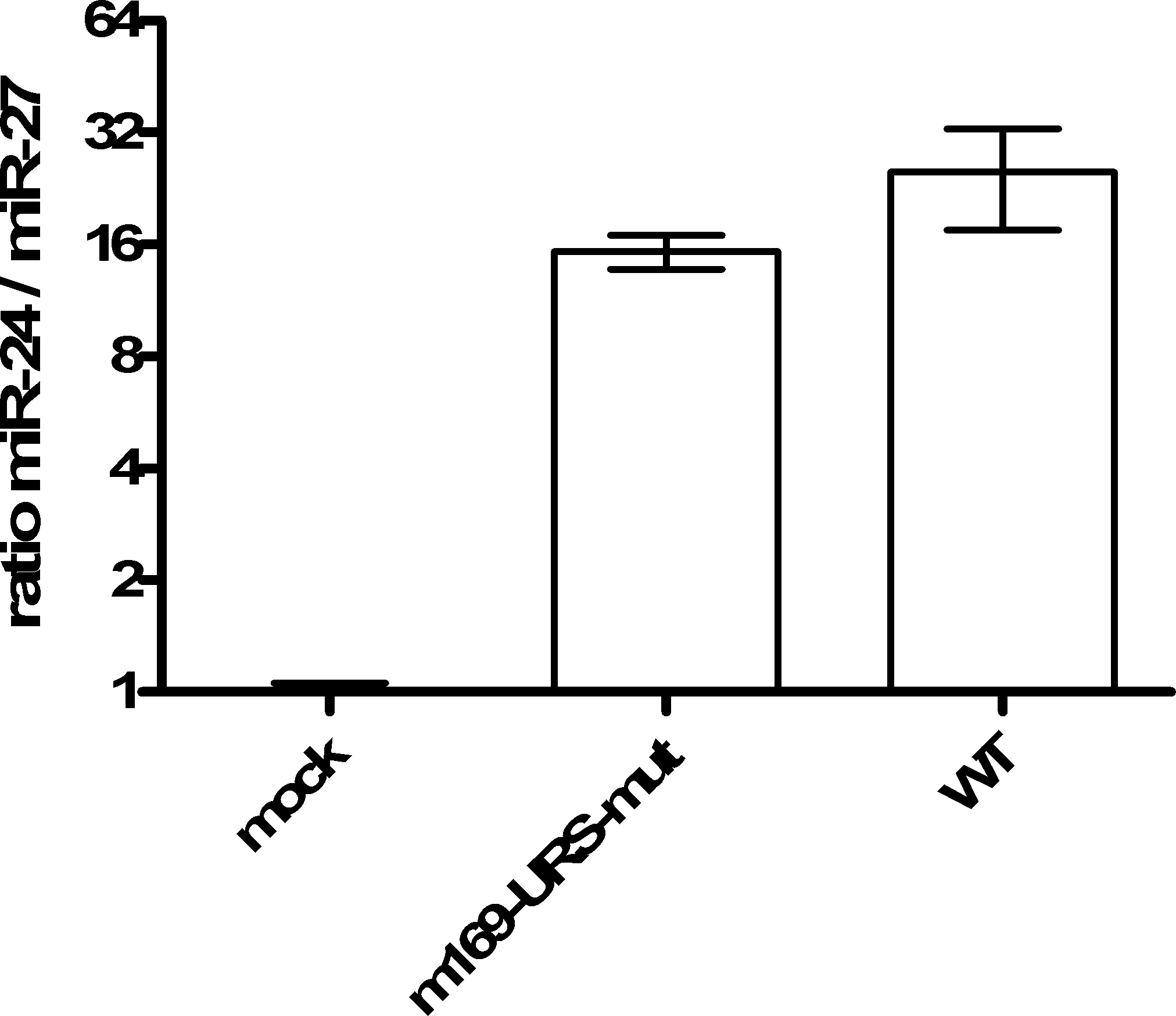

Supplement: Figure S3 — Mutagenesis of the pyrimidine-rich stretch close to the miR-27 binding site has no significant effect on miR-27 degradation. To generate MCMV-m169-URS-mut, the pyrimidine-rich stretch located 69 nt 3′ of the miR-27 binding site was mutated from ‘TTTTTGTTTTTT’ to ‘AAGAAGAAAGAA’ using markerless mutagenesis. The primers used to amplify the Kanamycin resistance gene encoded on the plasmid pEPKAN-S are included in Table S5. Following virus reconstitution, NIH-3T3 cells were infected with mock, m169-URS-mut or wild-type MCMV (WT) at an MOI of 10. At 48 hpi miR-24 and miR-27 levels were determined by q-RT-PCR. No significant difference in miR-27 degradation between m169-URS-mut and wild-type MCMV infection was observed. (TIF) [file ppat.1002510.s003.tif]
